# Legislative Debate-Attributed Suicidality Among LGBTQ+ Adults: The Buffering Effect of Community Belongingness

**DOI:** 10.3390/healthcare14020278

**Published:** 2026-01-22

**Authors:** Keith J. Watts, Shawndaya S. Thrasher, Laneshia R. Conner, Nicole Campbell, Louis G. Baser, DeKeitra Griffin, Sydney P. Howard, Missy Spears, Justin X. Moore

**Affiliations:** 1 College of Social Work, University of Kentucky, Lexington, KY 40508, USA; laneshia.conner@uky.edu; 2 Center for Health, Engagement, and Transformation, College of Medicine, University of Kentucky, Lexington, KY 40536, USA; sydney.howard@uky.edu (S.P.H.); jx.moore@uky.edu (J.X.M.); 3 School of Social Work, Louisiana State University, Baton Rouge, LA 70802, USA; sthrasher@lsu.edu (S.S.T.); dgrif36@lsu.edu (D.G.); 4 Darden College of Education & Professional Studies, Old Dominion University, Norfolk, VA 23529, USA; ncampbel@odu.edu; 5 College of Nursing, University of Kentucky, Lexington, KY 40536, USA; louisbaser3@uky.edu; 6 Queer Kentucky, Louisville, KY 40206, USA; missy@queerkentucky.com

**Keywords:** LGBTQ+, suicidality, structural stigma, legislative debates, belongingness, minority stress, intersectionality

## Abstract

**Background:** In recent years, the sociopolitical landscape in the United States has shifted due to an increase in state-level legislation regarding LGBTQ+ rights, a trend that has been particularly pronounced in the Commonwealth of Kentucky. While the mental health impacts of enacted laws are increasingly documented, a critical gap remains in understanding the psychological toll of the legislative debates themselves—the prolonged periods of public discourse surrounding the restriction of rights. **Methods:** Utilizing data from the 2025 Queer Kentucky Survey (*N* = 817), this exploratory study examined the association between LGBTQ+ community belongingness and acute suicidality attributed specifically to anti-LGBTQ+ legislative debates. Data were derived from a non-probability snowball sample. Binary logistic regression models that adjusted for age, race, gender identity, education, and income were utilized. **Results:** Prevalence of debate-attributed suicidality was alarmingly high: 59.7% of the sample attributed increased suicidal thoughts, and 44.1% attributed a suicide attempt, specifically to the legislative debates. LGBTQ+ belongingness was a robust protective correlate, associated with significantly lower odds of both suicidal thoughts (*OR* = 0.61, *p* < 0.001) and attempts (*OR* = 0.41, *p* < 0.001). Analyses further revealed divergent risk for suicidality across demographic characteristics. **Conclusions:** Findings are consistent with the interpretation that legislative debates may function as distinct structural stressors associated with suicidal thoughts and suicide attempts. While community belongingness may offer a critical buffer, the elevated risks among Transgender and Black, Indigenous, and People of Color (BIPOC) populations highlight the need for intersectional, structural interventions beyond individual resilience.

## 1. Introduction

### 1.1. The Legislative Landscape as a Determinant of Health

The United States is currently witnessing an unprecedented wave of legislation regarding the rights and existence of lesbian, gay, bisexual, transgender, and queer (LGBTQ+) individuals. The 2024 and 2025 legislative sessions have surpassed previous records for anti-LGBTQ+ activity, with the American Civil Liberties Union [1] and the Trans Legislation Tracker [2] documenting hundreds of bills introduced nationwide. This legislative surge has disproportionately targeted transgender and gender-expansive (TGE) individuals, with over 1000 anti-TGE bills focusing on healthcare bans, curriculum censorship, and public accommodation restrictions being introduced [3,4].

The Commonwealth of Kentucky has emerged as a focal point in this national legislative trend. Building on the 2023 enactment of a sweeping ban on gender-affirming care for minors [5], the 2025 legislative session advanced structural restrictions across multiple public sectors. In education and civic life, legislation was introduced to codify biological definitions of sex [6], restrict restroom access in public schools [7], and defund diversity, equity, and inclusion initiatives in higher education [8]. Simultaneously, the legislature targeted healthcare and carceral systems by proposing bans on Medicaid coverage for gender-affirming care [8], attempting to nullify protections against conversion therapy [9], and mandating biological sex separation and care denials within correctional facilities [10,11]. These policy actions represent political determinants of health that create risk environments for marginalized populations [12]. Researchers have increasingly framed such legislative environments as forms of structural stigma—societal-level conditions, cultural norms, and institutional policies that constrain the opportunities, resources, and well-being of the stigmatized [13,14].

### 1.2. The Legislative Process as an Ambient Stressor

While the deleterious effects of enacted laws are increasingly documented [15,16], a critical gap remains in understanding the psychological toll of the legislative debates themselves—the prolonged periods of public litigation over rights that often precede the passage of a bill. Recent scholarship suggests that the mere introduction and debate of these bills, regardless of their passage, creates a deleterious environment. Horne et al. [17] characterize this phenomenon as an ambient stressor that signals social devaluation and permeates the daily lives of LGBTQ+ individuals. This phenomenon, characterized by Dhanani and Totton [18] as legislative “rumination”, involves the chronic monitoring of news cycles and anticipatory anxiety regarding the loss of rights. Fenton et al. [19] found that the volume of proposed anti-LGBTQ+ legislation was significantly associated with poor mental health among college students, even in states where bills did not pass. This suggests that the political debate itself operates as a distal stressor, creating a climate of hostility that impacts mental health independent of legal outcomes [20,21]. Recent research on a nationally representative sample of high school students reinforces this, finding that state-level policies regarding sexual orientation are significantly associated with suicide planning [22].

### 1.3. Theoretical Framework: An Intersectional Sociological Approach

**Figure 1:**
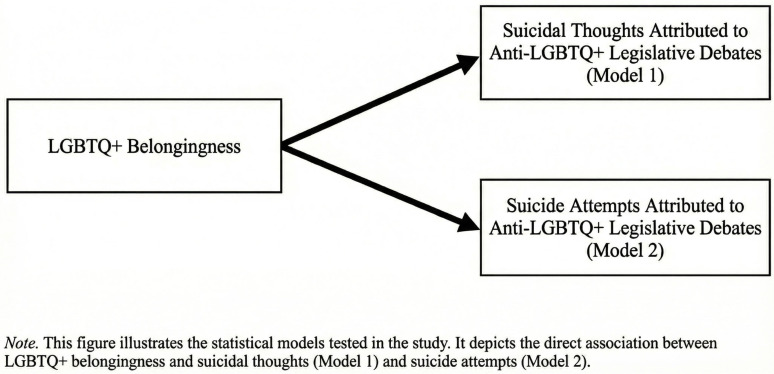
Theoretical Model of LGBTQ+ Belongingness and Suicidality Attributed to Anti-LGBTQ+ Legislative Debates.

To understand the psychological sequelae of this political climate, this study utilizes an intersectional socioecological framework [23] as a guiding paradigm. Within this multi-level context, we integrate Minority Stress Theory [24], Baumeister and Leary’s Belongingness Hypothesis [25], and the Interpersonal Theory of Suicide (IPTS) [26] to explicate the mechanisms linking structural stigma to individual suicidality (see Figure 1).

Minority Stress Theory posits that structural stigma acts as a distal stressor that penetrates the individual psyche, increasing vigilance and internalization of stigma [27]. Legislative debates operate as acute environmental microaggressions, delivering a steady stream of messages that define LGBTQ+ identities as socially undesirable or invalid. Central to our analysis is the understanding of belongingness not merely as a social amenity, but as a fundamental human motivation. Baumeister and Leary [25] argue that the need to belong is a powerful, innate drive to form and maintain lasting, positive relationships. Structural stigma, particularly legislative debates regarding the validity of LGBTQ+ identities, may directly undermine both components. By threatening the legal recognition of relationships (e.g., marriage) and fostering a climate of hostility that makes daily interactions fraught with potential conflict, these debates may systematically thwart the fundamental need to belong.

The IPTS extends this by identifying the specific mechanisms of suicidality, which include Thwarted Belongingness (the painful sense of disconnection) and Perceived Burdensomeness (the belief that one is a liability to others) [26]. It follows that anti-LGBTQ+ debates, which often frame queer people as a public safety threat [28], may exacerbate thwarted belongingness on a macro scale by signaling exclusion from the civic participation. Furthermore, the rhetoric surrounding parents’ rights [29] and the protection of children may engender perceived burdensomeness, as LGBTQ+ individuals internalize the message that their existence imposes a moral or social burden on their families and the state [30].

### 1.4. The Protective Role of Belongingness

Conversely, community belongingness—the internal sense of connection and solidarity with other LGBTQ+ people—may help buffer this stress [31,32]. Distinct from general social support, LGBTQ+ specific belongingness provides a unique form of identity-specific scaffolding that validates the self in the face of structural erasure [33,34]. According to the buffering hypothesis, high levels of social connection may interrupt the pathway between discrimination and adverse mental health outcomes [35]. Yet, the role of belongingness is complex; emerging research suggests it may function differently across intersections of race and gender, sometimes failing to protect against the compounded effects of racism and transphobia [36].

### 1.5. Current Study

Despite the growing body of literature on structural stigma, research specifically examining the psychological toll of legislative debates in high-risk, rural regions like Kentucky remains sparse. Furthermore, few studies have simultaneously tested the protective role of belongingness in the context of such acute political hostility. To address these gaps, the current study utilizes data from the 2025 Queer Kentucky Survey to examine the association between LGBTQ+ belongingness and acute suicidality attributed to legislative debates. Attributing suicidality directly to the debates allows for an assessment of the specific impact of the political environment, distinct from baseline suicidality. Specifically, this exploratory study aims to: (1) quantify the prevalence of debate-attributed thoughts and attempts of suicide among LGBTQ+ adults in Kentucky, and (2) determine if LGBTQ+ belongingness is associated with lower odds of these outcomes. We hypothesized that the prevalence of debate-related suicidality would be high given the increasingly hostile political climate (H1). Consistent with the theory that satisfying the need to belong is associated with reduced pathology [25], we further hypothesized that higher levels of LGBTQ+ belongingness would be significantly associated with lower odds of both increased suicidal thoughts and suicide attempts attributed to Anti-LGBTQ+ legislative debates (H2).

## 2. Materials and Methods

### 2.1. Participants and Procedure

Data for this study were drawn from the 2025 wave of the Queer Kentucky Survey, an annual, community-informed cross-sectional assessment of LGBTQ+ adults residing in Kentucky [37]. Recruitment occurred between 8 April and 17 July 2025, using non-probability convenience and snowball sampling strategies [38,39]. Snowball sampling was selected to facilitate access to isolated individuals in rural or high-stigma environments who may not be connected to formal LGBTQ+ organizations. This strategy is particularly effective for reaching populations where visibility carries potential safety risks [40,41]. We acknowledge, however, that this non-probability approach limits generalizability and may overrepresent individuals who are more connected to LGBTQ+ networks or those experiencing higher levels of psychological distress regarding the legislative climate.

To access a diverse cross-section of the population, the study leveraged digital channels, including social media, email listservs, and the nonprofit’s website, alongside in-person recruitment at community events such as Pride festivals and health fairs. Potential participants were directed to an online survey platform (Qualtrics) where they reviewed an electronic informed consent document prior to participation. To ensure safety and transparency, a content warning advised respondents that the instrument contained sensitive inquiries regarding mental health, substance use, and sexual behaviors. Due to the non-probability convenience and snowball sampling strategies utilized, a formal response rate could not be calculated. Eligibility was restricted to adults identifying as part of the LGBTQ+ community living in Kentucky. As an incentive, completers could opt into a random drawing for one of twenty-five $100 gift cards. The study protocol received ethical approval from the University of Kentucky Institutional Review Board (Protocol #94156), and the final analytic sample consisted of 817 participants. The sample was predominantly young adults (18–25), reflecting the generational demographics of LGBTQ+ identification and the specific vulnerability of emerging adults to political climates [42].

### 2.2. Measures

#### 2.2.1. LGBTQ+ Belongingness

The primary independent variable was LGBTQ+ belongingness. This 18-item LGBTQ+ Belongingness Attainment Scale (BAS) [33] was utilized for its ability to distinguish internal psychological belongingness from mere behavioral participation, assessing multiple facets of belonging, including connectedness, affiliation, and companionship within the LGBTQ+ community. Specific, identity-based measures of belongingness have been shown to have greater predictive validity for minority mental health than generic social support scales [43,44]. Participants responded to statements such as “You feel a sense of connectedness to the LGBTQ+ community”, “You feel the problems and challenges of the LGBTQ+ community have an impact on you”, and “It is important for you to participate in LGBTQ+ community events and activities”. Responses were recorded on a 6-point Likert-type scale ranging from “Strongly Disagree” (1) to “Strongly Agree” (6). Items were coded such that higher scores consistently reflected greater belongingness, and a composite score was calculated by computing the mean of the 18 items (*M* = 4.79, *SD* = 1.02). The BAS has previously demonstrated strong psychometric properties within this specific sample population [37], and has demonstrated excellent internal consistency (α = 0.95) in the current study.

#### 2.2.2. Debate-Attributed Suicidal Thoughts

Participants were asked how much debates about anti-LGBTQ+ legislation increased their thoughts of suicide using a 4-point ordinal scale that included “A lot”, “Some”, “A little”, and “Not at all”. For the primary analysis, responses were dichotomized into a binary outcome. Affirmative response was coded as 1 (“Yes”) and “Not at all” was coded as 0 (“No”).

#### 2.2.3. Debate-Attributed Suicide Attempts

Participants were asked how much debates about anti-LGBTQ+ legislation were associated with suicide attempts using a 4-point ordinal scale that included “A lot”, “Some”, “A little”, and “Not at all”. For the primary analysis, responses were dichotomized into a binary outcome. Affirmative response was coded as 1 (“Yes”) and “Not at all” was coded as 0 (“No”).

#### 2.2.4. Demographic Variables

To account for structural and identity-based differences, demographic variables were recoded into broader categories for analysis. Due to sample size constraints for specific age groups, participants were categorized into two age cohorts: young adults (18–30 years; 62.8%) and adults (31–60 years; 37.2%). Similarly, due to sample size constraints for specific racial groups, race was dichotomized into White or Caucasian (73.3%) and Black, Indigenous, and People of Color (BIPOC), which included Black/African American, Asian, American Indian/Alaska Native, and multiracial participants (26.7%). Gender identity was collapsed into three groups: Cisgender (39.3%), Transgender (18.2%), and Non-binary/Gender fluid/Not listed (42.5%). Education was dichotomized as having a high school diploma/GED or less (44.1%) versus a college or professional degree (55.9%). Finally, annual household income was categorized into four categories: $50,000 or less (36.1%), $50,000–$75,000 (20.6%), $75,000–$100,000 (21.3%), and greater than $100,000 (22.1%). Full demographic characteristics for the analytic sample are presented in Table 1.

### 2.3. Data Analysis

All statistical analyses were conducted using SPSS version 31. Descriptive statistics were first computed to characterize the sample. Subsequently, two separate binary logistic regression models were constructed to evaluate the association between LGBTQ+ belongingness and the two adverse mental health outcomes. Prior to modeling, outcome variables were dichotomized. This decision was driven by the clinical imperative to identify the presence of suicide risk rather than subjective gradations of intensity, as any attribution of suicidal intent to political determinants represents a critical risk marker requiring intervention. Furthermore, dichotomization facilitates the calculation of clear odds ratios, offering more interpretable effect sizes for policy advocacy. Both models were adjusted for age, race, gender identity, education, and income. Approximately 12% of the total sample was excluded from the final models due to missing data, resulting in a final analytical sample of *n* = 722 for the suicidal thoughts model and *n* = 720 for the suicide attempts model.

Univariate and bivariate analyses were conducted to describe the sample and examine unadjusted associations between variables. Bivariate associations were assessed using Pearson correlations for continuous variables and point-biserial correlations for associations involving binary variables. Multicollinearity among independent variables was assessed using Variance Inflation Factors (VIF), utilizing a threshold of 2.5 to identify potential concerns [45,46]. Additionally, the linearity of the logit assumption was assessed for the continuous belongingness predictor using the Box-Tidwell procedure. Finally, logistic regression model fit was evaluated using a combination of calibration and discrimination metrics. While the Hosmer–Lemeshow goodness-of-fit test was calculated, interpretations prioritized indices less sensitive to sample size, such as Nagelkerke’s pseudo-*R*^2^, Tjur’s Coefficient of Discrimination (*R*^2^), and the Area Under the Curve (AUC). Methodological literature suggests that the Hosmer–Lemeshow test frequently indicates poor fit in larger samples due to the detection of practically negligible discrepancies between observed and predicted risks [47,48]. Therefore, we assessed model performance primarily using discrimination metrics and variance explained [49,50]. Results are reported as adjusted odds ratios (*OR*) with 95% confidence intervals. Statistical significance was established at an alpha level of 0.05.

## 3. Results

### 3.1. Descriptive Statistics and Bivariate Associations

Descriptive statistics for the analytic sample are presented in Table 1. The sample demonstrated a relatively high mean level of community belonging, with an average score on the LGBTQ+ Belongingness Scale (BAS) of 4.79 (Range = 1–6; *SD* = 1.02). A majority of participants (59.7%, *n* = 479) reported attributing increased suicidal thoughts specifically to the debates around anti-LGBTQ+ and anti-trans legislation. Furthermore, nearly half of the sample (44.1%, *n* = 353) reported having attempted suicide attributed to this sociopolitical climate.

Bivariate correlations among all study variables are presented in Table 2. Consistent with the study’s hypotheses, LGBTQ+ belongingness was significantly and negatively correlated with both increased suicidal thoughts (*r* = −0.28, *p* < 0.001) and suicide attempts (*r* = −0.44, *p* < 0.001), indicating that higher levels of community connection were associated with lower reports of debate-related suicidality. A strong positive correlation was also observed between the two outcome variables, with increased suicidal thoughts strongly associated with suicide attempts (*r* = 0.58, *p* < 0.001). Regarding demographic factors, age showed a protective pattern; older participants (31–60) were significantly less likely to report both suicidal thoughts (*r* = −0.15, *p* < 0.001) and attempts (*r* = −0.14, *p* < 0.001) compared to emerging adults. Gender identity also emerged as a significant correlate in bivariate analyses; identifying as transgender or gender expansive (TGE) was positively correlated with both suicidal thoughts (*r* = 0.11, *p* = 0.002) and attempts (*r* = 0.14, *p* < 0.001), and negatively correlated with belongingness (*r* = −0.27, *p* < 0.001), suggesting that TGE individuals experienced higher distress and lower community connection than their cisgender LGBQ peers.

### 3.2. Multivariable Predictors of Debate-Attributed Suicidality

Two binary logistic regression models were estimated to determine the unique contribution of LGBTQ+ belongingness to suicidality outcomes after adjusting for age, race, gender identity, education, and income (Table 3).

#### 3.2.1. Model Diagnostics and Fit

Diagnostics indicated that the assumptions for logistic regression were met. Multicollinearity was not present, as VIF values for all predictors ranged from 1.01 to 1.25, falling well below thresholds of concern [45,46]. Evaluation of model fit suggested robust predictive capability. Although the Hosmer–Lemeshow test was statistically significant for both the suicidal thoughts model (*p* = 0.032) and the suicide attempts model (*p* < 0.001)—a finding consistent with the test’s known sensitivity to large sample sizes [47,48]—alternative metrics demonstrated adequate to excellent fit [49]. The model for suicidal thoughts showed acceptable discrimination (AUC = 0.72) and explained a significant proportion of variance (Nagelkerke *R*^2^ = 0.18; Tjur *R*^2^ = 0.14). Similarly, the model for suicide attempts demonstrated excellent discrimination (AUC = 0.80) and variance explanation (Nagelkerke *R*^2^ = 0.32; Tjur *R*^2^ = 0.26).

#### 3.2.2. Predictors of Suicidal Thoughts

The first model, predicting increased suicidal thoughts, was statistically significant (χ^2^ (9, *N* = 722) = 101.86, *p* < 0.001). LGBTQ+ belongingness was significantly associated with lower odds of the suicidal thoughts. For every one-unit increase in belongingness, the odds of reporting increased suicidal thoughts decreased by 39% (*OR* = 0.61, *p* < 0.001, 95% CI [0.51, 0.74]). Demographic covariates also revealed significant disparities. Age and education were associated with lower odds of suicidal thoughts: older adults (31–60) had significantly lower odds of suicidal thoughts compared to emerging adults (*OR* = 0.61, *p* = 0.004, 95% CI [0.43, 0.85]), and those with a college degree or higher had lower odds than those with a high school education or less (*OR* = 0.54, *p* < 0.001, 95% CI [0.37, 0.78]). Notably, participants identifying as transgender had more than double the odds of reporting increased suicidal thoughts compared to cisgender participants (*OR* = 2.31, *p* < 0.001, 95% CI [1.41, 3.77]). Racial identity was not a statistically significant predictor of suicidal thoughts in this model (*p* = 0.338).

#### 3.2.3. Predictors of Suicide Attempts

The second model, predicting suicide attempts attributed to the debates, was also statistically significant (χ^2^(9, *N* = 720) = 199.98, *p* < 0.001). LGBTQ+ belongingness was significantly associated with lower odds of suicide attempts; a one-unit increase in belongingness was associated with a 59% decrease in the odds of reporting a suicide attempt (*OR* = 0.41, *p* < 0.001, 95% CI [0.34, 0.51]). While age (*OR* = 0.51, 95% CI [0.35, 0.74]) and education (*OR* = 0.44, 95% CI [0.30, 0.65]) continued to show protective associations similar to the first model, the risk profile regarding race and gender shifted significantly. Unlike the model for thoughts, gender identity was not a significant predictor of suicide attempts (*p* = 0.142). Instead, BIPOC participants had significantly higher odds of reporting a suicide attempt compared to White participants (*OR* = 2.12, *p* < 0.001, 95% CI [1.44, 3.13]). Additionally, participants in the $75,000–$100,000 (*OR* = 1.72, *p* = 0.031, 95% CI [1.05, 2.83]) and $100,001+ (*OR* = 1.65, *p* = 0.046, 95% CI [1.01, 2.70]) income brackets had significantly higher odds of attempts compared to those in the lowest income bracket.

## 4. Discussion

### 4.1. The Toll of Legislative Debates

This study suggests that the legislative debates surrounding anti-LGBTQ+ bills in Kentucky may function as a potent structural stressor. It is critical to contextualize the reported prevalence rates: 59.7% of participants reported increased suicidal thoughts, and 44.1% reported a suicide attempt that they specifically attributed to these debates. These figures represent subjective attributions within a non-probability community sample and should not be interpreted as population-level psychiatric prevalence rates. However, the magnitude of this self-reported distress aligns with IPTS, suggesting that the legislative climate may thwart belongingness and increase perceived among LGBTQ+ constituents. By focusing on the debate process rather than just enacted laws, our findings illuminate how the ambient stress of structural stigma permeates the daily lives of queer Kentuckians, potentially operating as a distinct mechanism of harm.

### 4.2. Belongingness as a Buffer

Consistent with our hypothesis, LGBTQ+ belongingness emerged as a significant protective correlate. As levels of belongingness increased, the odds of attributing suicidal thoughts and attempts to the debates decreased significantly. This finding supports the buffering hypothesis within Minority Stress Theory, indicating that connection to a community of shared fate may help insulate individuals from macro-level hostility [27]. In the context of the IPTS, fostering community connection may counteract the thwarted belongingness generated by state-sanctioned exclusion, potentially preventing the progression from distress to suicidal desire [26].

### 4.3. Disparities by Gender, Race, and Income

Our results revealed divergent risk profiles that validate the utility of the intersectional socioecological framework [23] applied in this study. By examining risk across multiple axes of identity, we found distinct patterns that a single-axis analysis would have obscured. Transgender participants had significantly higher odds of suicidal thoughts compared to cisgender peers, a finding that reflects the specific targeting of transgender existence in recent bills (e.g., bathroom bans, healthcare restrictions). This legislative scrutiny likely produces a profound sense of identity invalidation specific to gender diversity.

Conversely, BIPOC participants showed significantly higher odds of suicide attempts despite showing no significant difference in suicidal thoughts. While this study did not directly measure individuals capability for suicide [51], this disparity is theoretically consistent with literature suggesting that systemic racism may function as a habituating painful and provocative event [52,53]. Repeated exposure to racialized trauma may increase the capability to enact lethal self-harm, potentially accelerating the transition from ideation to attempt among BIPOC LGBTQ+ individuals [52]. We offer this as a theoretical framework for interpreting the data, acknowledging that further research is needed to empirically test this pathway.

Additionally, an unexpected exploratory finding emerged regarding socioeconomic status: participants in higher income brackets ($75k–$100k+) had significantly higher odds of suicide attempts compared to those with the lowest income. This result is counterintuitive given the established protective effect of SES in general population samples. It may reflect unique stressors for high-SES LGBTQ+ individuals in conservative regions, such as greater professional visibility or fear of status loss. However, given the exploratory nature of this finding, it should be interpreted with caution and warrants replication in future probability samples.

### 4.4. Limitations

Several limitations must be acknowledged. First, the cross-sectional design precludes causal inference; while participants explicitly attributed their suicidality to legislative debates, we cannot rule out bidirectional relationships between distress and political vigilance. Furthermore, data collection occurred almost immediately following the 2025 Kentucky legislative session. This temporal proximity likely heightened the salience of these debates, potentially inflating prevalence rates compared to non-legislative periods.

Second, the use of convenience and snowball sampling may overrepresent individuals who are already connected to LGBTQ+ networks [39]. As an exploratory investigation utilizing non-probability sampling, these findings represent a signal of distress within a specific community network rather than a population-wide prevalence estimate. This is particularly relevant in rural Kentucky, where invisibility is often a necessary survival strategy, potentially leading to an underestimation of risk among the most isolated [54]. Third, suicidality was assessed using single-item measures specifically attributing thoughts and attempts to legislative debates. While this prevents direct comparison with general clinical prevalence rates, it captures the distinct construct of sociopolitical stress attribution, which is critical for understanding the specific impact of the legislative environment. Furthermore, we acknowledge that suicidal thoughts are conceptually harder to capture and predict due to issues of recall bias and operational fluidity, whereas suicide attempts represent more concrete, tangible behavioral endpoints. This distinction likely contributed to the difference in model performance, where the included predictors explained a substantially larger proportion of variance for attempts compared to thoughts.

Fourth, regarding missing data, while the use of non-probability sampling introduces inherent selection bias, Little’s MCAR test confirmed that item-level missing data within the sample were missing completely at random. This suggests that the exclusion of cases via listwise deletion did not introduce further systematic bias into the regression models. Finally, the dichotomization of demographic variables—while necessary for statistical power—masks within-group heterogeneity, particularly among the diverse racial and gender identities grouped together. Additionally, the use of a general LGBTQ+ belongingness measure may obscure the nuanced experiences of participants possessing additional minoritized identities, who may derive different protective benefits from belonging to other identity-specific communities (e.g., racial or gender-specific) compared to the broader LGBTQ+ community.

### 4.5. Implications for Practice and Policy

These findings underscore that legislative activity is a substantial social determinant of mental health. Clinicians working with LGBTQ+ clients in hostile political climates cannot treat anxiety and suicidality as purely intrapsychic phenomena; they must explicitly assess the impact of sociopolitical stress. We recommend that intake procedures in these regions include questions regarding clients’ exposure to legislative news cycles and their perceived safety. Therapeutic interventions should focus on validating the rationality of this distress—helping clients externalize the source of their anxiety to the structural environment rather than internalizing it as personal pathology. Furthermore, given the disparities found among transgender and BIPOC participants, clinicians must practice with cultural humility, recognizing that for multiply marginalized clients, the “political” is often immediately and dangerously personal.

The finding that LGBTQ+ belongingness significantly lowers the odds of suicidality identifies community connection as a primary mechanism of suicide prevention. Consequently, LGBTQ+ community centers, pride organizations, and digital spaces should not be viewed merely as social clubs, but as critical public health infrastructure. Funding bodies and state health departments should prioritize the financial sustainability of these organizations, particularly in rural areas where the survival strategy of invisibility [54] hinders access to organic support networks.

Ultimately, clinical and community interventions are downstream solutions to an upstream problem. Public health advocacy must target the root cause of this distress: the proliferation of legislation that institutionalizes stigma. Public health officials should call for Health Impact Assessments to be attached to proposed anti-LGBTQ+ bills, formally documenting the potential psychiatric costs of such legislation before it comes to a vote. By framing legislative debates not just as policy discourse but as matters of public safety, advocates can leverage these data to demonstrate that the *process* of debating these rights endangers the lives of Kentucky’s citizens.

### 4.6. Future Directions

Future research should utilize longitudinal designs to track mental health across the legislative cycle to better establish temporal causality. Researchers should also employ more granular intersectional analyses to unpack the specific mechanisms driving the high attempt rates among gender- and racial-minoritized groups. This aligns with Standley’s [23] argument that effective suicide prevention must move beyond individual pathology to interrogate the structural power dynamics that condition life and death.

Additionally, future studies should further investigate the exploratory finding regarding household income. The unexpected association between higher income and suicide attempts may reflect unique stressors faced by LGBTQ+ individuals in high-earning professional environments, such as heightened exposure to political scrutiny or discriminatory professionalism [55]. This suggests that socioeconomic visibility in certain sociopolitical climates may carry increased risk, a hypothesis that warrants testing in probability samples. Furthermore, future interventions, such as clinical trials and community-based participatory research, must account for the differential suicide experiences linked to intersectional identities. Participants with multiple marginalized identities may exhibit distinct behavioral patterns of suicidality that require tailored, culturally responsive prevention strategies.

## 5. Conclusions

The findings of this study suggest that the legislative debates surrounding anti-LGBTQ+ bills in Kentucky may not be benign political processes but rather potential structural stressors with severe consequences. The high prevalence of debate-attributed suicidality indicates a public health crisis driven by state-sanctioned stigma. However, the potent protective association of belongingness offers a clear avenue for intervention. As Baumeister and Leary [25] presciently argued, the drive to form and maintain relationships is fundamental to survival. In a legislative environment that moves to codify their marginalization, the ability of LGBTQ+ individuals to forge deep, psychological connections with one another serves as a vital, life-saving resource. Public health efforts must therefore prioritize the preservation and funding of the community infrastructures that make such belonging possible.

## Figures and Tables

**Table 1:** Sample Characteristics and Key Variables (N = 817).

Characteristic	*n*	%
Age (*n* = 766)		
18–25	243	31.7
26–30	238	31.1
31–35	167	21.8
36–40	63	8.2
41–60	55	7.2
Race/Ethnicity (*n* = 801)		
White or Caucasian	587	73.3
Black or African American	174	21.7
American Indian/Alaska Native	24	3.0
Asian	16	2.0
Gender Identity (*n* = 812)		
Non-binary/Gender Fluid	282	34.7
Cisgender Woman	190	23.4
Cisgender Man	129	15.9
Transgender Man	97	11.9
Transgender Woman	51	6.3
Not Listed	63	7.8
Sexual Orientation (*n* = 813)		
Queer	290	35.7
Gay	137	16.9
Lesbian	133	16.4
Bisexual	123	15.1
Pansexual	43	5.3
Asexual/Demisexual	43	5.3
Heterosexual/Straight	44	5.4
Education Level (*n* = 810)		
Less than High School	9	1.1
High School Diploma or GED	348	43.0
College Degree	318	39.3
Professional Degree	135	16.7
Annual Household Income (*n* = 793)		
<$25k	87	11.0
25k–$49,999	199	25.1
$50k–$74,999	163	20.6
$75k–$99,999	169	21.3
≥$100k	175	22.1
Suicidality		
Suicidal Thoughts	479	59.7
Attempted Suicide	353	44.1
	***M***	***SD***
LGBTQ+ Belongingness	4.79	1.02

Note. Granular demographic data are presented for descriptive purposes; variables were collapsed into broader categories (e.g., 18–30) for regression analyses to ensure sufficient cell counts. Valid percentages are reported to account for item-level missing data. Total *N* varies slightly by variable due to missing responses.

**Table 2:** Descriptive Statistics and Bivariate Correlations.

Variable	M (SD)	α	1	2	3	4	5	6	7	8
1. Suicidal Thoughts	0.61 (0.49)		—							
2. Attempted Suicide	0.45 (0.50)		0.58 **	—						
3. LGBTQ+ Belongingness	4.76 (1.03)	0.95	−0.28 **	−0.44 **	—					
4. Age	1.73 (0.96)		−0.15 **	−0.14 **	−0.29 **	—				
5. Race	1.27 (0.44)		−0.01	0.03	−0.09 *	−0.03	—			
6. Education	1.56 (0.50)		−0.24 **	0.16 **	0.40 **	0.10 **	0.11 **	—		
7. Income	2.31 (1.17)		−0.13 **	−0.01	0.14 **	0.21 **	0.06	0.29 **	—	
8. Gender Identity	2.04 (0.90)		0.11 **	0.14 **	−0.27 **	−0.12 **	−0.04	−0.16 **	−0.08 *	—

Note. Coefficients are Pearson correlations and point-biserial correlations for binary variables. *p* < 0.05 *, *p* < 0.01 **.

**Table 3:** Binary Logistic Regression Models Predicting Suicidality.

	**Model 1: Suicidal Thoughts**				
**Predictor**	**B**	**SE**	***p***	**OR**	**95% CI**
Constant	3.29 **	0.52	<0.001	—	—
LGBTQ+ Belongingness	−0.49 **	0.10	<0.001	0.61	[0.51, 0.74]
Age (31–60)	−0.50 **	0.17	0.004	0.61	[0.43, 0.85]
Race (BIPOC)	−0.18	0.19	0.338	0.83	[0.57, 1.21]
Education (College Degree+)	−0.62 **	0.19	<0.001	0.54	[0.37, 0.78]
Income (Ref: <$50k)					
$50k–$75k	0.15	0.24	0.520	1.17	[0.73, 1.85]
$75k–$100k	−0.03	0.24	0.911	0.97	[0.61, 1.55]
$100k+	−0.38	0.24	0.113	0.69	[0.43, 1.09]
Gender Identity (Ref: Cisgender)					
Transgender	0.84 **	0.25	<0.001	2.31	[1.41, 3.77]
Non-binary/Gender fluid	0.08	0.19	0.649	1.09	[0.76, 1.57]
Model Fit					
Nagelkerke *R*^2^	0.18				
Tjur’s *R*^2^	0.14				
*χ*^2^ (df = 9)	101.86 **		<0.001		
AUC	0.72				
	**Model 2: Attempted Suicide**				
**Predictor**	**B**	**SE**	***p***	**OR**	**95% CI**
Constant	4.18 **	0.54	<0.001	—	—
LGBTQ+ Belongingness	−0.89 **	0.10	<0.001	0.41	[0.34, 0.51]
Age (31–60)	−0.68 **	0.19	<0.001	0.51	[0.35, 0.74]
Race (BIPOC)	0.75 **	0.20	<0.001	2.12	[1.44, 3.13]
Education (College Degree+)	−0.82 **	0.20	<0.001	0.44	[0.30, 0.65]
Income (Ref: <$50k)					
$50k–$75k	0.09	0.25	0.719	1.10	[0.67, 1.80]
$75k–$100k	0.55 *	0.25	0.031	1.72	[1.05, 2.83]
$100k+	0.50 *	0.25	0.046	1.65	[1.01, 2.70]
Gender Identity (Ref: Cisgender)					
Transgender	0.37	0.25	0.142	1.44	[0.89, 2.35]
Non-binary/Gender fluid	−0.02	0.21	0.906	0.98	[0.65, 1.46]
Model Fit					
Nagelkerke *R*^2^	0.32				
Tjur’s *R*^2^	0.26				
*χ*^2^ (df = 9)	199.98 **		<0.001		
AUC	0.80				

Note. OR = odds ratio; CI = confidence interval; AUC = area under curve. All models adjusted for age, race, gender, education, and income. Reference categories: age = 18–25; race = White; education = High School/GED or less. *p* < 0.05 *, *p* < 0.001 **.

## Data Availability

The data presented in this study are available on request from the corresponding author. The data are not publicly available due to privacy restrictions.

## Abbreviations

The following abbreviations are used in this manuscript:

LGBTQ+	Lesbian, Gay, Bisexual, Transgender, Queer, and other sexual and gender identities
TGE	Transgender and Gender-Expansive
BIPOC	Black, Indigenous, and People of Color
IPTS	Interpersonal Theory of Suicide
BAS	Belongingness Attainment Scale
OR	Odds Ratio
CI	Confidence Interval
HB	House Bill
DEI	Diversity, Equity, and Inclusion
VIF	Variance Inflation Factor
